# Continuous versus intermittent enteral nutrition in hospitalised dogs and cats using a new scoring system: A prospective clinical study

**DOI:** 10.17221/26/2023-VETMED

**Published:** 2023-06-27

**Authors:** Aneta Rado-Blozonova, Leona Rauserova-Lexmaulova, Lucia Cerna, Matej Pavlik, Michal Rado, Michal Fusek

**Affiliations:** ^1^Small Animal Clinic, Faculty of Veterinary Medicine, University of Veterinary Sciences Brno, Brno, Czech Republic; ^2^Department of Physiology, Faculty of Veterinary Medicine, University of Veterinary Sciences Brno, Brno, Czech Republic; ^3^Department of Mathematics, Faculty of Electrical Engineering and Communication, Brno University of Technology, Brno, Czech Republic

**Keywords:** complications, nutritional score, nutritional support, outcome, tube feeding

## Abstract

Nutrition is an important part of the critical care. The goals of this prospective clinical study were to create a scoring system for the assessment of patient nutritional status and to compare continuous and intermittent bolus feeding of enteral nutrition (EN). A total of 38 patients (21 dogs and 17 cats) were divided into Group C (continuous feeding; 23 patients) and Group I (intermittent feeding; 15 patients). The following variables were recorded for each patient in both groups: gastrointestinal (GI) complications, changes in body weight (BW), body condition score (BCS), muscle condition score (MCS), veterinary nutritional status score (VetNSS), length of hospitalisation and outcome. The normality of the data was assessed using the Shapiro-Wilk test. Fisher’s exact test, Mann-Whitney, Wilcoxon signed-rank tests, and the *t*-test were used in statistical analyses. Significant changes were found in VetNSS and BW between the 1^st^ and 5^th^ day in Group C. The VetNSS seems to be useful for monitoring the nutritional status of hospitalised patients. Anorectic dogs and cats can be successfully supported with either continuous or intermittent feeding methods with a similar risk of GI complications and outcomes.

Ensuring optimal nutrition for critically ill patients has a fundamental role in their therapy and affects their recovery as well as overall survival. The body’s metabolic response to a severe illness, together with malnutrition has been associated with increased morbidity and mortality (Chan 2020; Schuetz et al. 2021). Therefore, in human medicine, there are a number of screening tools and scoring systems used for the early detection of risk factors of malnutrition and assessment of the overall nutritional status of patients, such as Controlling Nutritional Status (CONUT) score (de Ulibarri et al. 2005). The anthropometric parameters, history (such as weight loss, body mass index, triceps skin fold, appetite), and biomarkers (such as albumin, prealbumin, cholesterol and lymphocyte count) are usually used for nutritional assessment (Keller 2019). Only a few veterinary studies deal with the determination of biomarkers and risk factors of malnutrition in dogs (Michel 1993; Fabretti et al. 2015; Fabretti et al. 2021). However, no study to the author’s knowledge has focused on the development of a nutritional scoring system or other rating scale incorporating both clinical and laboratory parameters to provide an objective nutritional assessment in dogs and cats.

The enteral nutrition (EN) via feeding tubes is a common method of nutrition in both human and veterinary patients and could be provided as continuous or intermittent bolus feeding. Based on human medicine guidelines (Singer et al. 2019), we can assume that a continuous feeding method may be more suitable for critically ill patients, but intermittent feeding is a more physiological form of food intake. Human and veterinary studies did not determine which delivery method is more beneficial in providing prescribed calories and in terms of the incidence of gastrointestinal (GI) complications (Serpa et al. 2003; MacLeod et al. 2007; Campbell et al. 2010; Holahan et al. 2010; De Lazzaro et al. 2022).

The first aim of this study was to propose a Veterinary Nutritional Status Scoring System which could be useful for more accurate assessment and comparison of patient condition during feeding. The second aim was to compare continuous and intermittent EN in terms of incidence of GI complications, changes in body weight (BW), body condition score (BCS), muscle condition score (MCS), length of hospital stay and outcome in dogs and cats hospitalised at the intensive care unit.

## MATERIAL AND METHODS

Dogs and cats of all ages and weight categories requiring EN support via feeding tubes (nasoesophageal, nasogastric, oesophagostomy tube) admitted to the Intensive Care Unit of the University of Veterinary Sciences Brno, Czech Republic, were recruited from January 2018 through January 2021. Patients were followed for 5 days from the start of feeding in this study. Patients were excluded if the length of hospitalisation did not exceed at least 5 days or patients recovered, died or were euthanized during the reference period, placement of feeding tube was contraindicated, correction of blood parameters included in veterinary nutritional status score (VetNSS) was necessary such as in case of hypoglycaemia, hypophosphatemia, hypoalbuminemia, anaemia or owner consent was not obtained.

The type of feeding tube was determined based on the patient’s diagnosis whereas the size was chosen regarding the patient’s body weight. The correct position of nasoesophageal tubes was checked by negative aspiration and nasogastric tube by positive aspiration of gastric content from the tube and by administration of 5 ml to 10 ml of sterile saline into the tube without cough induction (Mathews 2017). The right lateral survey radiograph was performed to check the right oesophagostomy tube position in the distal third of the oesophagus (Mathews 2017).

Dogs and cats were divided into the intermittent (Group I) or continuous feeding group (Group C) based on their diagnosis and individual tolerance of the volume of EN. Patients in Group C received EN by continuous infusion (via syringe pump) while patients in Group I by intermittent bolus (via syringe every 4 h to 6 h). Resting energy requirements (RER) were calculated according to each patient’s current body weight (kg) by the following formula:

$$\text{RER}=70\times \text{BW}{\left(\text{kg}\right)}^{0.75}$$ (1)

Patients received a 1 kcal/ml, 1.5 kcal/ml veterinary liquid diets or mixed canned veterinary diets (Royal Canin Veterinary Liquid Diets, Royal Canin Veterinary Diet Cat/Dog Recovery, Hill’s Prescription Diet Canine/Feline A/D) according to the type of feeding tube and diagnosis. Feeding was initiated at 1/3 or 1⁄5 RER depending on the length of each patient’s anorexia (with anorexia > 5 days we started at 1⁄5 RER), then increased by 1/3 or 1⁄5 RER increments every 24 h until full RER was reached.

In patients, intolerant to the administration of a veterinary liquid or mixed canned diet with the onset of recurrent vomiting or regurgitation after feeding, the stomach size and fluid content was checked by ultrasound and intermittent gastric aspiration was performed via nasogastric tube. Moreover, the diet was replaced for the next 12–24 h by a mixture of a balanced isotonic infusion solution with glucose and a solution of vitamins, minerals and amino acids – trickle flow feeding (Kirby and Linklater 2017).

Veterinary Nutritional Status Scoring System was set up and we used a 7-variable model, which contained BCS, MCS, creatine kinase, albumin, glucose, phosphorus and haematocrit (Table 1).

**Table 1:** Veterinary Nutritional Status Scoring System for dogs and cats

DOGS						
Points						
3	2	1	0	1	2	3
1	2	3	BCS 4–5	6–7	8	9
4	3	2	MCS 1			
			CK 1–4	5–8	9–12	> 13
< 9	10–14	15–22	ALB 23–34	35–38	39–45	> 46
< 1.9	2–2.4	2.5–3	GLC 3.1–6.7	6.8–10	11–18	> 19
< 0.2	0.3–0.6	0.7–0.9	PHOS 1–2.1	2.2–3	3.1–4.5	> 4.6
< 19	20–29	30–34	HCT 35–56	53–59	60–65	> 66
CATS						
Points						
3	2	1	0	1	2	3
1	2	3	BCS 4–5	6–7	8	9
4	3	2	MCS 1			
			CK 1–4	5–8	9–12	> 13
< 9	10–14	15–22	ALB 23–35	36–39	40–45	> 46
< 1.9	2–2.4	2.5–3	GLC 3.1–6.9	7–14	15–20	> 21
< 0.2	0.3–0.6	0.7–0.8	PHOS 0.9–2	2.1–3	3.1–4.5	> 4.6
< 14	15–24	25–29	HCT 30–54	55–59	60–65	> 66

ALB = albumin (g/l); BCS = body condition score; CK = creatine kinase (μkat/l); GLC = glucose (mmol/l); HCT = haematocrit (%); MCS = muscle condition score; PHOS = phosphorus (mmol/l)

For MCS, a numerical scale of 1 to 4 was utilised, with scores of 1, 2, 3, and 4 corresponding to normal muscle mass status, mild muscle loss, moderate muscle loss and severe muscle loss, respectively.

The veterinary nutritional status score (VetNSS) was read on a point scale from 0 to 3, where zero points included values in the reference range. The further away the values of the monitored parameters were from the reference range, the more points were counted. The individual points assigned to each parameter were added to the final score of each patient. The higher result of the patient score means a worse metabolic status and the greater risk of developing malnutrition was anticipated. The patient nutritional status score was calculated for each patient at admission and fifth day of hospitalisation and both values were compared.

### Statistical analysis

The normality of the data was assessed using the Shapiro-Wilk test. Moreover, Q-Q plots and correspondence between the normalized histogram and the normal distribution density were assessed. The normality of age, body weight, length of the hospital stay, BCS and MCS was rejected at the significance level of 0.05.

The only exception was VetNSS where the normality was not rejected. On that account, the Mann-Whitney test (Welch’s two-sample *t*-test, respectively) was used for the comparison of non-normally (normally, respectively) distributed parameters between groups. In order to compare BW, BCS, MCS and VetNSS within groups, the Wilcoxon signed-rank test and the paired *t*-test were used. All the calculations were carried out in R (R Core Team 2020). Between-group differences in sex, mortality and the incidence of GI complications were compared using Fisher’s exact test in MedCalc statistical software v17.2 (MedCalc Software Ltd., Belgium). A significance level of 0.05 was used in all the analyses.

## RESULTS

**Figure 1:**
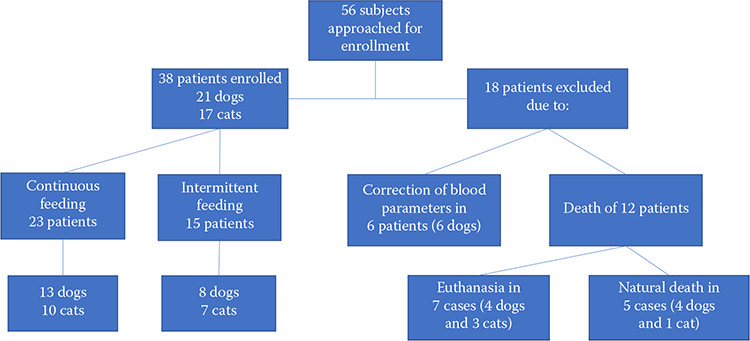
Population characteristics of dogs and cats enrolled in the study

Fifty-six animals were considered in the study, of which 38, twenty-one dogs and seventeen cats, met all criteria for data analysis and 18 patients were excluded (Figure 1).

The age of patients in Group C ranged from 1 to 16 years with a median of 7 years (7.3 ± 3.9), and patients in Group I ranged from 3 months to 14 years with a median of 9 years (6.8 ± 5.1). No significant difference in age was found between the groups (*P* = 0.79). There were 10 patients with esophagostomy (all in Group I) and 28 with nasoesophageal/nasogastric feeding tubes (23 in Group C and 5 in Group I).

No significant differences between Groups C and I were found in BW, BCS, MCS, length of hospital stay and outcome (Table 2).

**Table 2:** Outcome data of 38 canine and feline patients admitted for data analysis

Variables	Group C	Group I	*P*-value	Test
BW (kg) at admission	4.7 (2.2–41.5) 8.987 (10.142)	5 (2–37) 8.990 (10.263)	1.00	M-W
BW (kg) on the 5^th^ day	4.5 (2.1–39.6) 8.649 (9.683)	4.9 (2.15–38.5) 9.063 (10.961)	0.82	
Delta BW (admit to discharge)	–0.2 (–2.4–0.5) –0.338 (0.669)	–0.1 (–1.5–2.5) 0.073 (0.905)	0.31	
BCS at admission	5(3–7) 4.739 (1.096)	5 (2–7) 4.667 (1.234)	0.86	
BCS on the 5^th^ day	5 (3–7) 4.652 (1.152)	5 (2–7) 4.600 (1.242)	0.89	
MCS at admission	2 (1–3) 1.913 (0.848)	1 (1–3) 1.467 (0.743)	0.10	
MCS on the 5^th^ day	2 (1–3) 1.957 (0.825)	1 (1–3) 1.600 (0.737)	0.19	
Length of hospital stay (days)	8 (3–15) 9.130 (3.571)	9 (4–28) 9.867 (6.186)	0.88	
				
VetNSS at admission	5 (1–10) 5.957 (2.345)	5 (0–8) 4.600 (2.197)	0.08	T
VetNSS on the 5^th^ day	5 (1–11) 4.739 (2.750)	2 (0–9) 3.467 (2.875)	0.19	
				
GI complications	9	3	0.31	F
Mortality – total deaths	8 (34.8%)	4 (33.3%)	0.61	

Data reported as a median (minimum, maximum range) on the first row and a mean (standard deviation) on the second row; comparison of data between Group C and I using the Mann-Whitney test (M-W), the *t*-test (T) and the Fisher’s exact test (F)

BCS = body condition score; BW = body weight; GI complications = gastrointestinal complications; MCS = muscle condition score; VetNSS = veterinary nutritional status score

When comparing the values between the 1^st^ and 5^th^ day of hospitalisation, there was a significant decrease in BW (*P* = 0.02) and in the VetNSS (*P* = 0.03) in Group C. In 13/23 patients the scoring system values decreased during hospitalisation, in 9/23 patient values increased and in 1/23 the value was the same. The VetNSS also decreased in 9/15 patients from Group I, but the change was not significant.

Sixteen percent of patients (6/38, 3 dogs and 3 cats) vomited, 16% (6/38, 5 dogs and 1 cat) patients developed regurgitation and 5% of patients (2/38, only cats) were affected by diarrhoea. Both, vomiting and regurgitation together, were reported in two patients (one cat and one dog). Patients who suffered from gastrointestinal problems before the placement of the feeding tube were not counted among patients with gastrointestinal complications during the study period.

There was no significant difference in the frequency of gastrointestinal complications during EN between Group C and I (Table 2). In dogs, there was an insignificantly higher frequency of regurgitation (5/21) than in cats (1/17, *P* = 0.2).

## DISCUSSION

Our study led to the fulfilment of both goals, firstly we created a Veterinary Nutritional Status Scoring System and found a significant change of VetNSS and BW during hospitalisation in patients fed continuously. Secondly, we did not find any significant changes in the frequency of GI complications, BW, BCS, MCS, length of hospital stay and outcome between both groups of patients.

The Veterinary Nutritional Status Scoring System was derived from the Acute Patient Physiologic and Laboratory Evaluation (APPLE) score, which is commonly used to quantify and assess disease severity, and was more focused on patient metabolic and nutritional parameters (Hayes et al. 2010; Hayes et al. 2011). We used a 7-variable model (BCS, MCS, creatine kinase, albumin, glucose, phosphorus, haematocrit) (Table 1). BCS and MCS provide a semiquantitative method of evaluating body fat and muscle mass (WSAVA Nutritional Assessment Guidelines Task Force Members et al. 2011). Body weight was not included in the scoring system, because it is influenced by hydration status, residual volume of the stomach, urinary bladder and oedema/effusion presence (You et al. 2013; Kirby and Linklater 2017). Blood parameters were selected because they are non-specific indicators of the body’s metabolic status and can be considered as indicators of malnutrition and associated complications. Creatine kinase is the most sensitive indicator of muscle damage in human patients (Vohanka 2012). During malnutrition, muscle catabolism and loss of muscle mass occur, so we can consider increased creatine kinase activity as an indirect indicator of malnutrition. In two veterinary studies, decreased haematocrit and hypoalbuminemia were associated with malnutrition and poor clinical outcome in critically ill patients (Michel 1993; Fabretti et al. 2015). One recent veterinary study did not show the importance of monitoring serum albumin alone as a marker of malnutrition, moreover, its level can be significantly affected by the inflammatory response (Fabretti et al. 2021). Hypophosphataemia in long-term anorectic patients is a cause of the development of haemolytic anaemia, which is part of the refeeding syndrome (RS) (Mathews 2017). Hyperglycaemia is another possible complication in long-term anorectic patients, when glucose intolerance develops (King et al. 2018).

A significant change in VetNSS during hospitalisation (from admission to day 5) was found only in patients fed continuously. The decreasing of the score probably resulted from an improvement in the metabolic status of patients in Group C and therefore a positive effect of continuous EN. However, VetNSS also insignificantly decreased in patients fed intermittently. It can be assumed that the improvement of the metabolic status occurred in both groups of patients and the statistical significance was influenced by a smaller number of patients in Group I.

Also, a significant change in BW during hospitalisation (from admission to day 5) was observed in Group C. However, body weight is a variable parameter influenced by, for example, patient hydration, GI tract and urinary bladder content (You et al. 2013). The importance of evaluating changes in body weight as a parameter itself in assessing the patient’s nutritional status is therefore questionable. As indicated by the significant change in VetNSS, the form of the scoring system is more suitable for evaluating the impact of nutrition.

There was no difference found in BW, BCS, MCS changes, length of hospitalisation and outcome between patients fed continuously and patients fed intermittently in this study. These results support the findings of other veterinary studies that compared different routes of EN in hospitalised patients (Brunetto et al. 2010; Holahan et al. 2010). No differences in GI complication rates were found between patients fed continuously and patients fed intermittently in this study. This finding supports the results of other veterinary and human studies (Serpa et al. 2003; MacLeod et al. 2007; Campbell et al. 2010; Holahan et al. 2010).

This study has some limitations, mainly the small population of the patients being followed, which can lead to difficulties in the statistical evaluation of the data (type II statistical error). Another limitation of this study was the initial heterogeneous patient population in terms of disease severity and overall health. It can be assumed that a number of monitored parameters, including overall survival, were significantly affected by the severity of the disease in individual patients. In one veterinary study, the disease severity was determined as the main negative factor on the outcome and also had a negative effect on energy intake (Brunetto et al. 2010). The calculation of incidence of GI complications was another limitation because some patients had evidence of vomiting, regurgitation, or diarrhoea before initiation of EN. Therefore, it is difficult to clearly distinguish GI complications due to underlying disease or the administration of EN. Moreover, patients with these symptoms before initiation of EN were not counted among patients with GI complications in this study and therefore the total number of patients with GI complications may be distorted.

The last limitation of this study was the division of patients into groups of continuous and intermittent feeding, which was performed based on their diagnosis, clinical condition and nutritional preferences evaluated by the clinician. Random distribution of patients would be more appropriate.

In conclusion, the Veterinary Nutritional Status Scoring System seems to be a good tool for improving the assessment of the metabolic status of patients and could also become a prognostic factor for the development of malnutrition and associated complications, thus reflecting the overall survival of patients. However, more attention should be paid to the overall energy intake of each hospitalised patient to prevent weight loss and nutritional status worsening. Anorectic dogs and cats can be successfully supported with either continuous or intermittent feeding methods with similar risks of GI complications and outcomes. Further investigation of the Veterinary Nutritional Status Scoring System and its prognostic value on a larger population of dogs and cats is warranted.
